# Disparate Effects of Lithium and a GSK-3 Inhibitor on Neuronal Oscillatory Activity in Prefrontal Cortex and Hippocampus

**DOI:** 10.3389/fnagi.2017.00434

**Published:** 2018-01-12

**Authors:** Tuan Nguyen, Theresa Fan, Susan R. George, Melissa L. Perreault

**Affiliations:** ^1^Department of Pharmacology and Toxicology, University of Toronto, Toronto, ON, Canada; ^2^Department of Medicine, University of Toronto, Toronto, ON, Canada

**Keywords:** GSK-3, lithium, neuronal oscillations, coherence, hippocampus, prefrontal cortex, spatial memory

## Abstract

Glycogen synthase kinase-3 (GSK-3) plays a critical role in cognitive dysfunction associated with Alzheimer’s disease (AD), yet the mechanism by which GSK-3 alters cognitive processes in other disorders, such as schizophrenia, remains unknown. In the present study, we demonstrated a role for GSK-3 in the direct regulation of neuronal oscillations in hippocampus (HIP) and prelimbic cortex (PL). A comparison of the GSK-3 inhibitors SB 216763 and lithium demonstrated disparate effects of the drugs on spatial memory and neural oscillatory activity in HIP and PL. SB 216763 administration improved spatial memory whereas lithium treatment had no effect. Analysis of neuronal local field potentials in anesthetized animals revealed that whereas both repeated SB 216763 (2.5 mg/kg) and lithium (100 mg/kg) induced a theta frequency spike in HIP at approximately 10 Hz, only SB 216763 treatment induced an overall increase in theta power (4–12 Hz) compared to vehicle. Acute administration of either drug suppressed slow (32–59 Hz) and fast (61–100 Hz) gamma power. In PL, both drugs induced an increase in theta power. Repeated SB 216763 increased HIP–PL coherence across all frequencies except delta, whereas lithium selectively suppressed delta coherence. These findings demonstrate that GSK-3 plays a direct role in the regulation of theta oscillations in regions critically involved in cognition, and highlight a potential mechanism by which GSK-3 may contribute to cognitive decline in disorders of cognitive dysfunction.

## Introduction

Glycogen synthase kinase-3 (GSK-3) is a constitutively active protein kinase with an array of physiological functions including mediating intracellular signaling, as well as regulating neuronal plasticity, gene expression, and cell survival (Grimes and Jope, 2001). GSK-3 exists as two isoforms, GSK-3α and GSK-3β, but the major research focus has been on GSK-3β as a result of its involvement in neuropsychiatric and neurodegenerative disease. Indeed, in recent years GSK-3 has been shown to be fundamental to the processes underlying cognitive function and evidence suggests that increased GSK-3 activity may represent a common biochemical mediator of impaired cognitive function in many central nervous system diseases (Emamian et al., 2004; Takashima, 2006; Ances et al., 2008; King et al., 2013).

A role for overactive GSK-3 as a critical mediator of cognitive decline in Alzheimer’s disease (AD) has been widely documented (Balaraman et al., 2006; Huang and Klein, 2006; Martinez and Perez, 2008; Muyllaert et al., 2008; Medina and Avila, 2010; Maqbool et al., 2016). In AD, overactivation of GSK-3 induces hyperphosphorylation of tau proteins resulting in the development of neurofibrillary tangles (NFTs) (Balaraman et al., 2006; Llorens-Martin et al., 2014) which are thought to contribute directly to cognitive dysfunction (Bloom, 2014). In schizophrenia, a disorder associated with cognitive dysfunction, a role for elevated GSK-3 activity in the neuropathology of this disorder is provided by postmortem findings, as well as genetic and behavioral studies. For example, reduced phosphorylation levels of GSK-3 (which correlate with increased activity) in postmortem frontal cortex samples of patients with schizophrenia has been documented (Emamian et al., 2004). In addition, associations between genetic variation in the GSK-3 gene and schizophrenia (SZ) have been reported (Li et al., 2011; Blasi et al., 2013; Tang et al., 2013) and a number of other susceptibility genes for SZ, including the genes encoding for the proteins disrupted in SZ (DISC1), neuregulin 1 (NRG-1), and dysbindin converge on GSK-3 signaling (Numakawa et al., 2004; Clapcote et al., 2007; Keri et al., 2009; Mao et al., 2009; Guo et al., 2010; Seres et al., 2010; Lipina et al., 2011, 2012). More recently, developmental inhibition of GSK-3 was shown to alleviate spatial memory deficiencies in a mouse model of schizophrenia predisposition (Tamura et al., 2016) indicative of a direct link between GSK-3 and the development of processes critical to aberrant cognitive functioning in this disorder. In line with these findings GSK-3 has been shown to be involved in mediating reductions in cognitive performance associated with diabetes mellitus (King et al., 2013; Wang and Zhao, 2016), Fragile-X syndrome (Guo et al., 2012; Franklin et al., 2014; Pardo et al., 2017), and human immunodeficiency virus (Ances et al., 2008), and in a model of traumatic brain injury improvements in spatial memory induced by valproate were associated with inhibition of GSK-3 in hippocampus (Dash et al., 2010).

A critical role for GSK-3 in inducing cognitive dysfunction via neurofibrillary tangle formation in AD is known. Yet, the involvement of this protein in other disorders of cognitive decline raises the possibility of an additional, and more direct, role for this kinase in mediating the processes underlying learning and memory not only in disease, but in healthy organisms as well. This idea is supported by studies in healthy animals showing that increased GSK-3 expression, within whole brain or in cortical and hippocampal neurons, could negatively affect cognitive function in both rats and mice (Hernandez et al., 2002; Liu et al., 2003). However, behavioral studies examining the effects of GSK-3 inhibitors on learning and memory under normal conditions are conflicting, with some reports showing positive (Nocjar et al., 2007; Lipina et al., 2013), negative (Al Banchaabouchi et al., 2004; Hu et al., 2009), or no effects of the drugs (Guo et al., 2012; Franklin et al., 2014). Despite these disparate findings, the large number of studies showing a role for GSK-3 in mediating cognitive performance in neurodegenerative disease, neuropsychiatric disorders and in injury, suggest that while a number of neuropathological processes may converge on GSK-3 signaling, the downstream effects of its increased activation are likely similar across many disease processes.

One possible consequence of increased GSK-3 activation is through the disruption of brain electrophysiological rhythms. Neuronal oscillatory rhythms, which are derived from macroscopic oscillations of neuronal populations, have patterns that are highly conserved across species (Buzsaki et al., 2013) and are critical to cognitive functioning (Igarashi, 2015; Helfrich and Knight, 2016). Neuronal oscillations in prefrontal cortex (PFC) and hippocampus (HIP) specifically have been shown to be of significant importance, having been linked with working memory in both humans (Cohen, 2011; Roux and Uhlhaas, 2014) and in animals (Pesaran et al., 2002; Benchenane et al., 2010; O’Neill et al., 2013), and dysregulated neuronal oscillatory activity in and between HIP and PFC have been widely documented in disease states such as AD and schizophrenia (Kwon et al., 1999; Herrmann and Demiralp, 2005; Schmiedt et al., 2005; Basar-Eroglu et al., 2007; Uhlhaas et al., 2008; Missonnier et al., 2010; Uhlhaas and Singer, 2010; Basar et al., 2013, 2016). For example, in humans or animal models reduced event-related increases in cortical gamma activity, or gamma-theta frequency coupling, in both AD and schizophrenia have been demonstrated (Kwon et al., 1999; Basar-Eroglu et al., 2007; Lodge et al., 2009; Missonnier et al., 2010; Zhang et al., 2016; Nakazono et al., 2017). Additionally in HIP, rodent models have demonstrated reduced elicited theta power, and/or gamma-theta coupling, in both disorders (Howlett et al., 2004; Villette et al., 2010; Scott et al., 2012; Mesbah-Oskui et al., 2015; Kalweit et al., 2017), with the augmentation of elicited theta power demonstrated to be characteristic of drugs used in clinical treatment of AD such as acetylcholinesterase inhibitors and memantine (Kinney et al., 1999; Guadagna et al., 2012).

Further evidence of a role for GSK-3 in neuronal oscillations comes from reports showing the regulation of ion channel function by this protein kinase (Wildburger and Laezza, 2012). Of relevance to the proposed study, GSK-3 has been shown to control the function of the *N*-methyl-D-aspartate (NMDA) receptor (Chen et al., 2007), a receptor also implicated in the regulation of oscillatory activity within the PFC and HIP (Lee et al., 2017; Lemercier et al., 2017; Miller et al., 2017). Specifically, Chen et al. (2007) demonstrated that the pharmacological inhibition or silencing of GSK-3 induced a long-lasting reduction of NMDA receptor-mediated ionic synaptic current in cortical pyramidal neurons. Interestingly, the effect of GSK-3 was specific for the GluN2B-containing NMDA receptors (Chen et al., 2007), receptors that are involved in the induction of long-term depression (LTD) (Liu et al., 2004; Massey et al., 2004). In addition to regulating NMDA receptor function, GSK-3 is also involved in voltage-gated sodium (Nav) channel function, both through regulation of surface expression (Yanagita et al., 2009; James et al., 2015) or functional activity via phosphorylation of fibroblast growth factor 14 (FGF14) (Shavkunov et al., 2013), a protein that interacts with the C terminus of Nav channels to regulate both the functional properties and subcellular distribution of the channels (Goldfarb et al., 2007; Shavkunov et al., 2013). Nav channels have been recently shown to play a role in the regulation of PFC and HIP gamma oscillations (Verret et al., 2012; Rama et al., 2015), and have been implicated as playing a key role in the neuropathophysiology of AD (Verret et al., 2012).

Lithium has been shown to inhibit GSK-3β directly *in vitro*, albeit with high Ki (∼2.0 for GSK-3β, Klein and Melton), and *in vivo* through activation of Akt (Beaulieu et al., 2004). However, lithium has also been demonstrated to inhibit other enzymes including inositol monophosphatases (IMPAs) (Berridge et al., 1989), bisphosphate 3′-nucleotidase (BPNT1) (Spiegelberg et al., 2005), and cyclooxygenase (COX) (Klein and Melton, 1996; Stambolic et al., 1996). Furthermore, lithium has been shown to influence numerous neurotransmitter systems including, serotonin, dopamine, and glutamate (Malhi et al., 2013). Despite the known role of GSK-3 in learning and memory, the effects of lithium on cognition are conflicting, with studies showing positive effects (Letendre et al., 2006; Nunes et al., 2013; Matsunaga et al., 2015; Daglas et al., 2016; Forlenza et al., 2016), little to no effect (Joffe et al., 1988; Schifitto et al., 2009; Bourne et al., 2013; Pfennig et al., 2014; Decloedt et al., 2016), or negative effects (Shaw et al., 1987; Monks et al., 2004; Senturk et al., 2007) of treatment on cognitive function. In the present study, we therefore sought to evaluate and compare the effects of a direct GSK-3 inhibitor, SB 216763, with lithium on the regulation of neuronal oscillatory activity within, and between, the HIP and PFC and the impact of these drugs on cognitive performance in a water maze test of spatial memory and reversal learning, tests that require HIP and PFC function, respectively (Broersen, 2000; Graybeal et al., 2011). Animals were administered five daily drug or vehicle injections with recordings taken from anesthetized rats at baseline, prior to behavioral testing on day 1, and following behavioral testing on day 1 and day 5.

## Materials and Methods

### Animals

Twenty-four adult male Wistar rats weighing approximately 350–400 g at the start of the experiments were used. Rats were housed up to three rats per cage in polyethylene cages in a colony room maintained on a 12-h light–dark cycle with free access to food and water. Rats were handled for 2 min daily for 5 days before the start of experiments. All treatments were performed during the light phase of the day–night cycle. All procedures involving animals complied with the guidelines described in the Guide to the Care and Use of Experimental Animals (Canadian Council on Animal Care, 1993), and were approved by the Animal Care Ethics Committee of the University of Toronto.

### Drugs

The GSK-3 inhibitor SB 216763 (Tocris Bioscience) was dissolved in a solution of DMSO, polyethylene glycol and sterile water, and administered at a dose of 2.5 mg/kg (i.p.) (Zhao et al., 2016; Wickens et al., 2017). Lithium chloride (lithium) was dissolved in 0.9% saline and administered at a dose of 100 mg/kg (i.p.). This dose was chosen as it was shown to increase phosphorylation of Akt (Zheng et al., 2013), an upstream negative regulator of GSK-3. For non-drug injections, an equivalent volume of vehicle (50% of the control animals received saline and 50% received DMSO, polyethylene glycol, sterile water) was administered. All injections were administered at a volume of 1.0 ml/kg.

### Behavior

Behavioral tests took place 10 min post-injection for SB 216763 and 30 min post-injection for lithium. Vehicle-treated animals were divided into two groups that underwent testing 10 or 30 min post-injection. For this group the data was pooled as no intra-group variation was evident. Animals were trained to locate a submerged platform in the Morris water maze using an allocentric task (i.e., using distal cues to find the platform) (Broersen, 2000). The maze consisted of a circular pool (2 m diameter) filled with water maintained at 23 ± 1°C. The pool contained a transparent circular platform (10 cm diameter) in one quadrant with the surface 3 cm below the water surface, and placed approximately 20 cm from the wall.

The allocentric task consisted of four trials per day with an inter-trial interval of 2 min for four consecutive days. Each trial began with placing the rat into the pool in one of the three quadrants that did not contain the platform (the starting quadrant was similar for all rats tested). Rats were allowed to swim for 2 min after which, if the rat did not find the platform, it was placed on the platform where it remained for a period of 30 s. For each subsequent trial the release point of the rat was shifted, however, the platform always remained in the same position. During the reversal, on day 5, the platform was moved to the quadrant opposite, and animals were allocated up to 15 trials to locate the platform under 20 s three times consecutively. Swimming trajectories were recorded and analyzed with Any-maze software (Stoelting). The escape latencies and distance traveled were obtained from each rat. The mean swimming distance and the mean daily latencies from the four daily trials were compared.

### Surgeries

Rats were anesthetized with isoflurane (induction 5%, maintenance 2%), administered the analgesic ketoprofen (5 mg/kg, s.c.) and secured in a stereotaxic frame. Body temperature was maintained at 37°C by a warming pad. Two custom length (25 mm) polyimide-insulated stainless steel wire twisted electrodes (Plastics One: E363/3-2TW, 0.125 mm) were implanted unilaterally into the dorsal CA1 region of the HIP (AP -3.6, ML -1.8, DV -2.9) and two into the prelimbic (PL) region of the PFC (AP +3.0, L -0.7, DV -4.0; Paxinos and Watson, 2005). Two screws attached to Teflon-insulated 20 mm length stainless steel wire (Plastics One, E363/20, 0.56 mm) were fixed into the skull above lambda as ground and the opposite hemisphere from electrode placement as reference. Additional anchor screws were attached to the skull and electrodes secured with dental cement to the anchor screws. The animals received an additional injection of ketoprofen 24 h following surgery and were allowed to recover individually in their home cage for a minimum of 10 days before the experiments were performed. Electrode placement was validated post-mortem.

### Electrophysiology

All LFP oscillatory recordings (Intan Technologies) were performed in anesthetized animals (isoflurane 2%) under a heating lamp. Breathing and heart rate were monitored while animals were under anesthesia. Baseline recordings were performed 3 days prior to the beginning of the behavioral experiment. During the behavioral experiment recordings were taken post-drug injection following (and not prior to) the last trial on Injection Day 1 (acute drug administration) or Injection Day 5 (repeated drug administration). We therefore chose to anesthetize the animals prior to recording to evaluate drug-evoked responses and at the same time eliminate the contribution of arousal state that may occur as a result of variations in overall swimming duration. LFP oscillatory data was collected for a 5 min period and sampled at a rate of 5000 samples/s. The spectral power of LFP oscillations in each region and coherence between regions were analyzed using routines from the Chronux software package for MATLAB (MathWorks). Recordings were downsampled, segmented, detrended, and low-pass filtered to remove high frequencies greater than 100 Hz. Continuous multitaper spectral power and coherence (tapers = [5 9]) between regions was calculated for each segment in the following frequency bands: delta (1–4 Hz), theta (>4–12 Hz), beta (>12–32 Hz), slow gamma (>32–59 Hz), and fast gamma (>61–100 Hz).

### Data Analysis

For the behavioral data, trials were averaged and the statistical significance of each dependent measure evaluated using a repeated measures ANOVA with a within subjects factor of Trial Day, and Drug as the between-subjects factors, and followed by Bonferroni *post hoc* tests. All data are expressed as means ± SEM. LFP spectral power was normalized to total spectral power. LFP power curves are reported as mean power for the slower frequencies and log-transformed for the gamma frequencies (>40 Hz) to better exhibit group differences. Quantification of the LFP power data at each frequency measure is reported as means ± SEM for between group comparisons, or as percent change from baseline for within group comparisons. For the within subjects analysis, a repeated measures ANOVA was performed for each frequency, with a within subjects factor of Injection # (baseline, 1 or 5 days), and followed by paired Student’s *t*-tests for *post hoc* comparisons to baseline. Some planned comparisons were performed to evaluate within subject changes between acute or repeated drug treatments with baseline. For the between subjects analysis, an ANOVA was performed for each frequency at each Injection # (baseline, 1, 5 days), with Drug as the between-subjects factor and followed by Bonferroni *post hoc* comparisons. Computations were performed using the SPSS/PC+ statistical package (IBM, Armonk, NY, United States).

## Results

### Effect of GSK-3 Inhibition on Spatial and Reversal Learning

**FIGURE 1:**
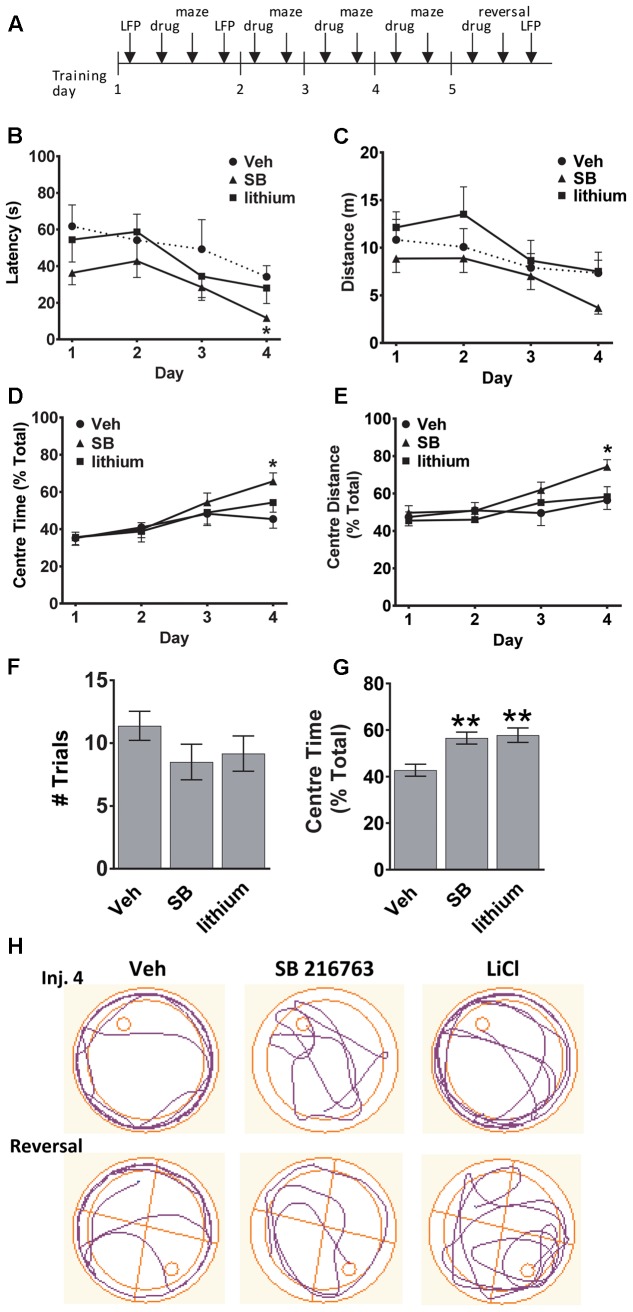
Effect of GSK-3 inhibition on learning and memory in the water maze. **(A)** Experimental timeline. **(B,C)** SB 216763 (2.5 mg/kg, i.p.), but not lithium (100 mg/kg, i.p.), reduced the latency to find the platform compared to vehicle controls by Injection Day 4, with no significant effects on the total distance traveled. **(D,E)** Rats treated with SB 216763 spent significantly more time, and traveled greater distance, in the center of the maze whereas vehicle-treated rats spent more time in the periphery. **(F)** There were no differences between and treatments on the number of trials required to find the platform during the reversal. **(G)** SB 216763 or lithium treatment resulted in rats spending more time in the center of the maze compared to the periphery during the reversal. **(H)** Representative swim plots from Injection 4 and the reversal test following drug treatment. *N* = 8 rats/group. Data are expressed as means ± SEM. ^∗^*p* < 0.05, ^∗∗^*p* < 0.01, compared to vehicle-treated rats, Bonferroni *post hoc* test.

The experimental timeline is depicted in **Figure 1A**. To evaluate whether GSK-3 inhibition could enhance spatial learning and memory the effects of selective GSK-3 inhibitor SB 216763, and the non-selective inhibitor lithium, on allocentric and reversal learning in the Morris water maze were examined. Significant differences between drug treatments in latency to find the platform and the distance traveled in the maze were evaluated. A comparison of all three groups across injections revealed a significant Drug × Trial Day interaction [*F*(6,60) = 2.5, *p* = 0.030]. SB 216763 resulted in improved spatial learning such that by Injection 4 animals that received SB 216763, but not lithium, exhibited a latency to escape significantly faster than the vehicle-treated controls (SB vs. Veh, *p* = 0.037, Bonferroni *post hoc*, **Figure 1B**) [Trial Day 4: Drug *F*(2,21) = 3.8, *p* = 0.041]. No significant differences in the total swimming distance were observed between groups (**Figure 1C**). Analysis of the swim trajectories revealed significant Drug × Trial Day interactions when Center Time [*F*(6,60) = 2.3, *p* = 0.038] and Center Distance [*F*(6,60) = 2.2, *p* = 0.04] were analyzed. By Trial Day 4, SB 216763 treatment resulted in the rats spending significantly more time in the center of the pool (SB vs. Veh, *p* = 0.023, Bonferroni *post hoc*, **Figures 1D,E**) which may reflect an anxiolytic effect of the drug (Crofton et al., 2017).

To assess cognitive flexibility, which requires PFC function (Graybeal et al., 2011), we next evaluated the animals during a reversal learning task by shifting the platform to the opposite quadrant and evaluating the number of trials it took to learn the new location of the platform and escape. No significant differences were evident between the three treatment groups (**Figure 1F**). Across all trials, both SB 216763- and lithium-treated animals spent a higher proportion of time searching the center of the maze for the platform during the reversal task (SB: 56.6 ± 2.6%, lithium: 57.8 ± 3.1%, Veh: 42.7 ± 2.6%, **Figure 1G**) [ANOVA: *F*(2,201) = 10.1, *p* = 0.0001]. Representative swim trajectories for Injection 4 and the reversal task are displayed in **Figure 1H**.

### Temporal Effects of GSK-3 Inhibition on HIP Oscillatory Activity

**FIGURE 2:**
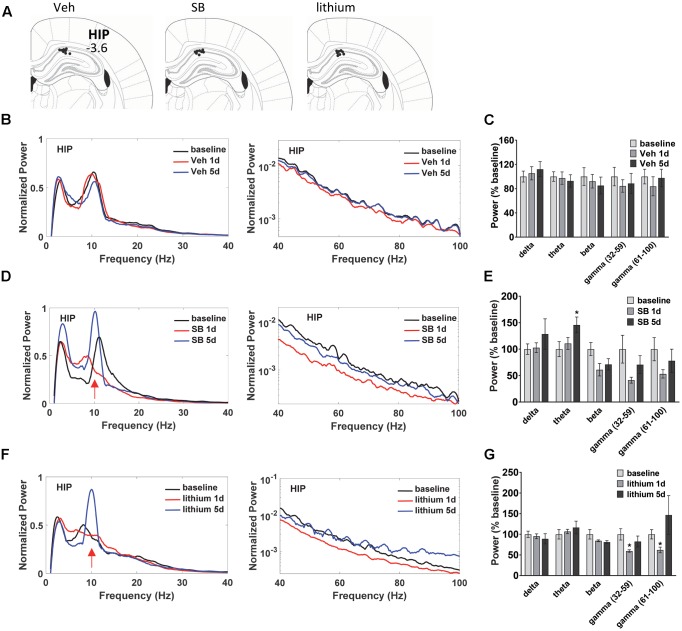
Temporal effects of GSK-3 inhibition on hippocampus (HIP) oscillations. **(A)** Electrode placements in HIP from each of the three treatment groups. **(B,C)** Power spectrums and quantification showing no deviations from baseline in HIP oscillatory power with vehicle administration. **(D)** Repeated GSK-3 inhibition by SB 216763 (2.5 mg/kg) induced a theta spike in HIP at approximately 10 Hz (red arrow) and induced an overall increased in theta power in this region. **(E)** Quantification of the HIP power spectrum at each frequency with SB 216763 treatment following water maze training on days 1 and 5, and at baseline. **(F,G)** Repeated lithium (100 mg/kg) induced a 10 Hz theta spike (red arrow) but did not induce an overall increase in theta power (4–12 Hz). Reduced slow and fast gamma powers were evident after acute treatment. *N* = 8 rats/group with 1–2 recordings/region/rat. Curves represent group means following acute (red) and repeated (blue) drug administration. Bars represent means ± SEM and are expressed as percent of baseline. ^∗^*p* < 0.05 compared to baseline, paired Student’s *t*-test.

Before the first swim on trial day 1, and following the final swim trial on days 1 and 5 LFP neuronal activity was evaluated in anesthetized animals to assess temporal changes in baseline, acute and repeated drug administration effects on oscillatory activity and coherence in and between dorsal HIP and PL (**Figure 2A**). LFPs are thought to represent the summed synchronous activity of relatively large groups of neurons (Mitzdorf, 1985; Logothetis, 2003), and can provide vital information on synchronous network activity both within and between regions. Evaluation of the oscillatory changes in HIP and PL of vehicle-treated rats (**Figures 2B,C**) during water maze training revealed no significant within subject Injection effects in HIP (**Figures 2B,C**). Recordings taken from SB 216763-treated animals (**Figures 2D,E**) showed that, in contrast to the vehicle-treated rats, SB 216763 treatment induced significant changes in HIP theta oscillations as revealed by a significant within subject effect of Injection [theta: *F*(2,22) = 4.6, *p* = 0.023]. Repeated, but not acute, SB 216763 increased theta power compared to baseline (*p* = 0.029, **Figures 2D,E**). There were no significant Injection effects of lithium treatment and planned comparisons to baseline revealed no significant effects on overall theta power (**Figures 2F,G**), however, both repeated SB 216763 or lithium administration induced a peak in the theta band at approximately 10 Hz compared to baseline, an effect also not evident in the vehicle-treated rats (**Figure 2B**). Acute lithium treatment suppressed both slow (*p* = 0.029 vs. baseline) and fast (*p* = 0.042 vs. baseline) gamma power in HIP, however, these effects were lost with repeated drug treatment (**Figures 2F,G**).

### Temporal Effects of GSK-3 Inhibition on PL Oscillatory Activity

**FIGURE 3:**
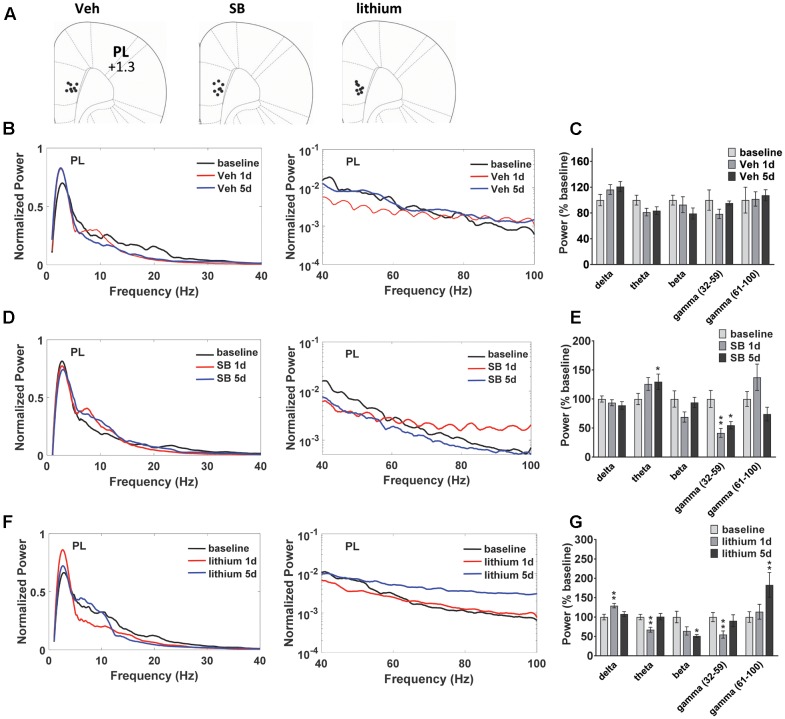
Temporal effects of GSK-3 inhibition on prelimbic cortex (PL) oscillations. **(A)** Electrode placements in PL from each of the three treatment groups. **(B,C)** Power spectrums and quantification showing no deviations from baseline in PL oscillatory power with vehicle administration. **(D)** Compared to baseline repeated SB 216763 (2.5 mg/kg) increased theta power in PL. Acute or repeated SB 216763 suppressed slow gamma power. **(E)** Quantification of the PL power spectrum at each frequency at baseline and following SB 216763 administration on days 1 and 5. **(F,G)** Changes in the power spectrum and quantification following acute or repeated lithium (100 mg/kg) treatment. *N* = 8 rats/group with 1–2 recordings/region/rat. Curves represent group means following acute (red) and repeated (blue) drug administration. Bars represent absolute value means ± SEM and are expressed as percent of baseline. ^∗^*p* < 0.05, ^∗∗^*p* < 0.01 compared to baseline, paired Student’s *t*-test.

Evaluation of drug effects in PL (**Figure 3A**) revealed no Injection effects following vehicle treatment (**Figures 3B,C**). Following SB 216763 administration an Injection effect was evident for the slow gamma band [*F*(2,24) = 9.325, *p* = 0.0001], with a reduction in slow gamma power following both acute and repeated drug treatment (**Figures 3D,E**). Similar to that observed in HIP, an increase in theta power was evident following repeated SB 216763 treatment (*p* = 0.033 vs. baseline). Animals treated with lithium exhibited significant Injection effects in the delta, theta, beta, and fast gamma bands [delta: *F*(2,30) = 7.6, *p* = 0.002; theta: *F*(2,30) = 9.4, *p* = 0.001; beta: *F*(2,30) = 6.2, *p* = 0.006; fast gamma: *F*(2,30) = 9.0, *p* = 0.001]. Compared to baseline acute lithium increased delta power (*p* = 0.001), and suppressed theta (*p* = 0.001), and slow gamma (*p* = 0.008) power, effects lost with repeated treatment. Following 5 days of lithium treatment animals exhibited reduced beta power compared to baseline (*p* = 0.011) and an increased in fast gamma power (*p* = 0.008, **Figures 3F,G**).

### Temporal Effects of GSK-3 Inhibition on HIP–PL Coherence

**FIGURE 4:**
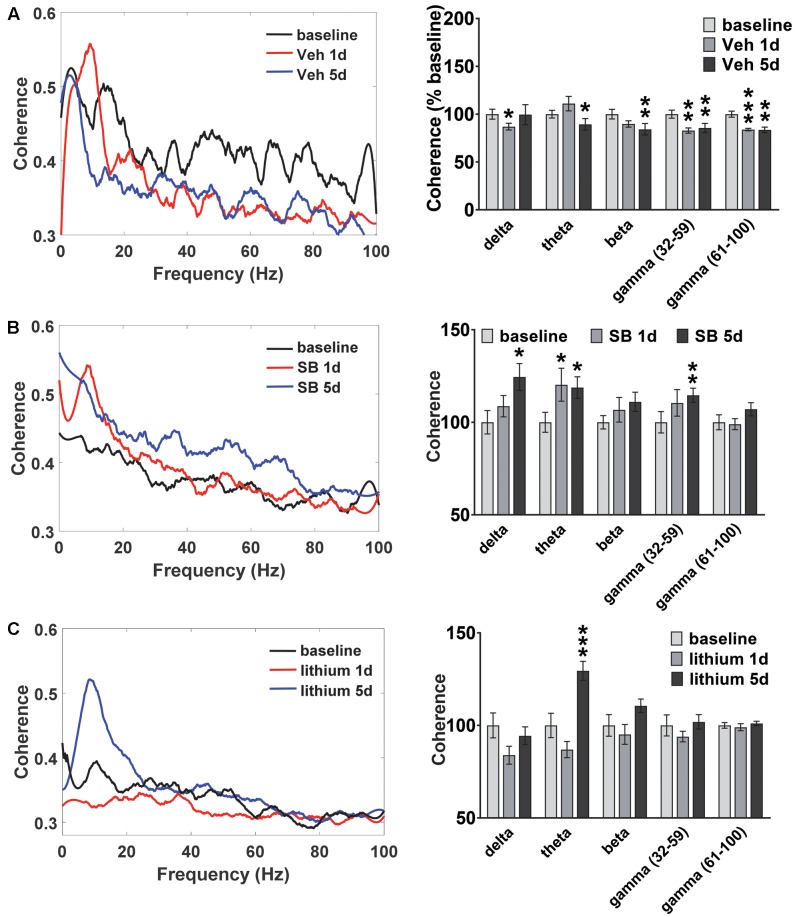
Temporal effect of GSK-3 inhibition on HIP-PL coherence. **(A)** Repeated vehicle administration suppressed HIP–PL coherence. **(B)** Acute or repeated SB 216763 (2.5 mg/kg) increased theta coherence compared baseline, with increased delta and low gamma coherence with repeated treatment. **(C)** Repeated lithium (100 mg/kg) increased theta power compared to baseline. *N* = 8 rats, 1–2 recordings/region/rat. Curves represent group means following acute (red) and repeated (blue) drug administration. Bars represent means ± SEM and are expressed as percent of baseline. ^∗^*p* < 0.05, ^∗∗^*p* < 0.01, ^∗∗∗^*p* < 0.001 compared to baseline, paired Student’s *t*-test.

Oscillatory coherence between HIP and PL in vehicle-treated rats and following GSK-3 inhibition was next examined (**Figure 4**). In vehicle-treated rats (**Figure 4A**) the repeated measures ANOVA revealed an Injection effect for all frequencies except delta [theta: *F*(2,22) = 5.3, *p* = 0.016; beta: *F*(2,22) = 3.6, *p* = 0.049; slow gamma: *F*(2,22) = 8.5, *p* = 0.002; fast gamma: *F*(2,22) = 13.9, *p* = 0.0001]. At the completion of the 5 days of water maze testing significant decreases in HIP–PL gamma coherence were observed for all frequencies compared to baseline (**Figure 4A**). Acute or repeated administration of SB 216763 increased HIP–PL coherence in the delta, theta and slow gamma bands (**Figure 4B**) [Injection Effects, delta: *F*(2,22) = 4.8, *p* = 0.018; theta: *F*(2,22) = 3.5, *p* = 0.048; slow gamma: *F*(2,22) = 3.5, *p* = 0.048]. Repeated, but not acute, lithium enhanced theta band power compared to baseline with no effects on the other frequencies (**Figure 4C**) [Injection Effects, theta: *F*(2,30) = 34.7, *p* = 0.0001].

### Effects of Direct GSK-3 Inhibition versus Lithium on HIP and PL Oscillations and Coherence

**FIGURE 5:**
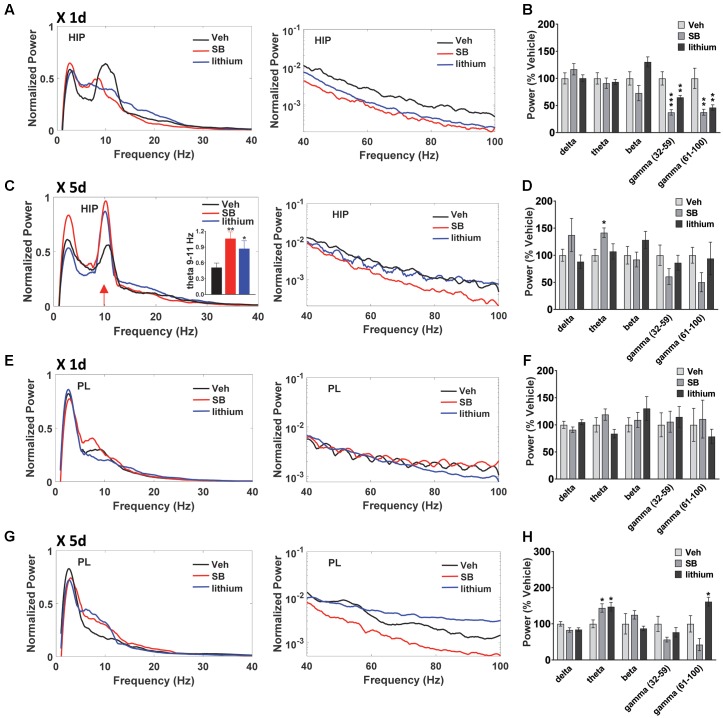
Effect of GSK-3 inhibition on HIP and PL oscillations. **(A,B)** Acute administration of SB 216763 (2.5 mg/kg) or lithium (100 mg/kg) reduced slow and fast gamma power in HIP. **(C)** Repeated SB 216763 or lithium induced a theta spike at approximately 10 Hz. **(D)** Quantification of the power spectrum showing increased overall theta power following SB 216763, but not lithium, compared to vehicle. **(E,F)** Acute SB 216763 or lithium did not alter PL oscillatory power. **(G,H)** Repeated SB 216763 or lithium increased theta power in PL compared to vehicle-treated rats. Lithium also induced an increase in fast gamma power. *N* = 8 rats/group with 1–2 recordings/region/rat. Curves represent group means following acute (red) and repeated (blue) drug administration. Bars represent means ± SEM. ^∗^*p* < 0.05, ^∗∗^*p* < 0.01, ^∗∗∗^*p* < 0.001 compared to vehicle, Bonferroni *post hoc* test.

**FIGURE 6:**
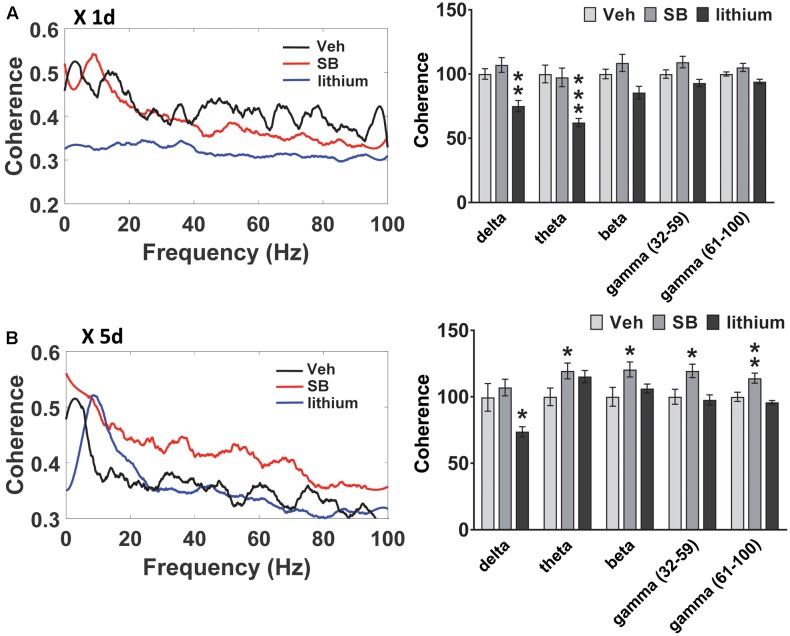
Effect of GSK-3 inhibition on HIP–PL coherence in rats. **(A)** No effect of acute SB 216763 (2.5 mg/kg) on HIP–PL coherence whereas lithium (100 mg/kg) suppressed delta and theta coherence. **(B)** Repeated SB 216763 increased HIP–PL coherence at all frequencies except delta. Lithium selectively suppressed delta coherence. *N* = 8 rats/group with 1–2 recordings/region/rat. Curves represent group means following acute (red) and repeated (blue) drug administration. Bars represent means ± SEM. ^∗^*p* < 0.05, ^∗∗^*p* < 0.01, ^∗∗∗^*p* < 0.001 compared to vehicle, Bonferroni *post hoc* test.

In addition to evaluating temporal changes in neural oscillatory power and coherence following drug treatment (within subjects comparisons), between group comparisons were also performed (**Figures 5**, **6**). In both HIP and PL, spectral power did not differ between groups prior to drug administration, nor did HIP–PL oscillatory coherence (Supplementary Figure S1). After a single injection of vehicle, SB 216763 or lithium, ANOVA analysis of spectral power revealed a significant effect of Drug in the beta, slow and fast gamma bands for HIP (**Figures 6A,B**) [beta: *F*(2,25) = 6.2, *p* = 0.005; slow gamma: *F*(2,25) = 15.8, *p* = 0.0001; fast gamma: *F*(2,25) = 8.5, *p* = 0.001]. Acute administration of either SB 216763 or lithium induced robust decreases in both slow and fast gamma compared to vehicle (**Figures 5A,B**). With repeated drug treatment significant Drug effects were evident in the theta and fast gamma bands [theta: *F*(2,25) = 3.5, *p* = 0.041; slow gamma: *F*(2,25) = 3.5, *p* = 0.039]. Compared to vehicle, only SB 216763 exhibited increased overall theta power following repeated treatment (**Figures 5C,D**). However, a significant increase in theta power (9–11 Hz) was evident following either SB 216763 (*p* = 0.005) or lithium (*p* = 0.028, **Figures 5C,D**) compared to the controls [ANOVA: theta (9–11), *F*(2,25) = 6.9, *p* = 0.003].

In PL no significant effects were evident following acute drug administration (**Figures 5E,F**), with significant theta and fast gamma Drug effects following repeated treatment [theta: *F*(2,25) = 5.4, *p* = 0.008; fast gamma: *F*(2,25) = 10.2, *p* = 0.0001]. Repeated SB 216763 or lithium each increased theta power compared to vehicle (*p* = 0.020 and *p* = 0.028, respectively, **Figures 5G,H**). Repeated lithium also increased fast gamma power in PL compared to vehicle-treated rats (*p* = 0.012).

A comparison of HIP–PL oscillatory coherence (**Figure 6**) between drug treatments revealed significant Drug effects at all frequencies [delta: *F*(2,25) = 8.3, *p* = 0.001; theta: *F*(2,25) = 2.9, *p* = 0.049; beta: *F*(2,25) = 4.0, *p* = 0.024; slow gamma: *F*(2,25) = 3.9, *p* = 0.024; slow gamma: *F*(2,25) = 3.4, *p* = 0.035]. Compared to vehicle, direct inhibition of GSK-3 by SB 216763 increased coherence at all frequencies except delta following repeated, but not acute treatment (**Figure 6**). Conversely, lithium suppressed HIP–PL delta and theta coherence with acute treatment, with reduced delta coherence with repeated treatment.

## Discussion

A pivotal role for GSK-3 in the regulation of learning and memory is widely accepted. Yet, except for its involvement in tau hyperphosphorylation and NFT formation in AD, little is known regarding the mechanisms by which GSK-3 regulates cognitive function. In the present study, we showed in rats that systemic inhibition of GSK-3 activity had direct effects on neuronal oscillatory activity in HIP and PL, and further, that direct GSK-3 inhibition induced oscillatory changes that were distinct from the non-selective GSK-3 inhibitor lithium. Selective inhibition of GSK-3 by SB 216763 significantly improved spatial memory and altered the animals’ learning strategy, increasing goal-directed exploratory behavior within the center of the maze. At the completion of behavioral testing, analysis of LFPs revealed there was a 10 Hz theta frequency spike in HIP following either SB 216763 or lithium. However, whereas selective GSK-3 inhibition increased overall theta power in both HIP and PL, lithium induced a theta power increase selectively in PL coincident with an increase in fast gamma power. Furthermore, whereas repeated SB 216763 increased HIP–PL coherence across all frequencies, lithium suppressed delta coherence, with no effect at the other frequencies.

Preclinical studies in animal models of disease have provided promising evidence of cognitive improvement as a result of treatment with GSK-3 inhibitors, with beneficial effects of GSK-3 inhibition, either by lithium or selective inhibitors, having been demonstrated in model systems of schizophrenia (Beaulieu et al., 2004; Chan et al., 2012; Lipina et al., 2013; Maksimovic et al., 2014), Fragile-X syndrome (Yuskaitis et al., 2010; Guo et al., 2012; Franklin et al., 2014; Pardo et al., 2017) as well as traumatic brain injury (Yu et al., 2012a,b). In addition, the large number of reports that have focused on AD, as a result of the known role of GSK-3 in AD neuropathology, are also positive showing significant improvements not only in cognitive performance but also in AD-related pathologies (Hu et al., 2009; Sereno et al., 2009; Gong et al., 2011; Onishi et al., 2011; Zhang et al., 2011; Avrahami et al., 2013; Noh et al., 2013; Nunes et al., 2015). In humans, however, the clinical efficacy of these compounds remains less clear, partly because of a lack of studies evaluating the impact of selective GSK-3 inhibitors on cognition and partly due to the large number of conflicting reports from studies evaluating the therapeutic effects of lithium. For example, clinical studies for the most part have demonstrated beneficial effects of lithium in improving cognition or preventing further cognitive decline in AD (Nunes et al., 2013; Matsunaga et al., 2015; Forlenza et al., 2016). Yet, positive effects (Letendre et al., 2006), or no effect (Schifitto et al., 2009; Decloedt et al., 2016) of lithium treatment have been shown for HIV-associated cognitive decline, and in bipolar disorder the findings are even more conflicting with reports showing positive effects (Daglas et al., 2016), little to no effects (Joffe et al., 1988; Bourne et al., 2013; Pfennig et al., 2014), or negative effects (Shaw et al., 1987; Monks et al., 2004; Senturk et al., 2007) of treatment on cognitive function.

In contrast to the numerous studies that have evaluated the effects of GSK-3 inhibition on cognition in neuropsychiatric and neurodegenerative disease, fewer studies have examined the effects of these compounds in healthy animals and humans. In animals, conflicting results due to differing dosing and treatment regimens make their impact on cognitive performance more difficult to define. For example, in mice acute treatment with the GSK-3 inhibitors TDZD-8 or VP0.7, or repeated treatment with SB 216763 every other day for 2 weeks, did not alter spatial learning (Guo et al., 2012; Franklin et al., 2014) whereas treatment with the dual phosphodiesterase-7 and GSK-3 inhibitor VP1.15 improved performance in the spatial object recognition test, the Y-maze task, and cued fear memory (Lipina et al., 2013). In rats, inhibition of GSK-3 following icv infusion of SB 216763 (20 ng/μl) did not affect performance in the Morris water maze (Tian et al., 2012), in contrast to the present findings which clearly showed a cognitive enhancing effect of the drug when repeatedly administered systemically at a dose of 2.5 mg/kg. Similar discrepancies have been reported with lithium with one study showing reduced spatial memory with intra-HIP administration (Parsaei et al., 2016), other studies showing enhanced cognitive performance with lithium treatment (Nocjar et al., 2007; Tsaltas et al., 2007), and other reports showing little or no effect of this compound in cognitive tasks (Vasconcellos et al., 2003; de Vasconcellos et al., 2005; Watase et al., 2007; Dai et al., 2012; Contestabile et al., 2013; King and Jope, 2013). The findings reported herein also showed a lack of effect of lithium in spatial learning using a dose previously shown to increase Akt activity (Zheng et al., 2013), a kinase known to phosphorylate and inhibit GSK-3, although positive trends were observed in some of the parameters evaluated. This lack of an effect of lithium on cognitive performance has also been consistently demonstrated in healthy humans, with some studies also showing lithium-induced mild impairments in various cognitive domains (Weingartner et al., 1985; Linnoila et al., 1986; Stip et al., 2000; Wingo et al., 2009).

The large number of conflicting reports evaluating the effects of lithium on cognition in both healthy and diseased states indicate that any of the observed therapeutic effects of lithium on cognition may not only reflect a combination of different dosing regimens but may be dependent on the disease under investigation. Although one mechanism of action of lithium is the inhibition of GSK-3, it is necessary to keep in mind that lithium has an array of biological substrates which would undoubtedly contribute to the drug’s effects. It is therefore logical that the functional effects of lithium could induce differing behavioral responses a result of interactions with the various neuropathologies under study. Lithium has been shown to inhibit other enzymes such as IMPAs (Berridge et al., 1989), BPNT1 (Spiegelberg et al., 2005), and cyclooxygenase (COX) (Klein and Melton, 1996; Stambolic et al., 1996). Therefore, even if lithium could have positive effects on cognitive processes through GSK-3 inhibition, some of these effects may be offset through its actions at other targets.

Although further research is required to determine the precise mechanisms underlying GSK-3 effects on cognition, one possible mediator is the NMDA receptor. In primary cortical pyramidal neurons or cortical slices GSK-3 was shown to phosphorylate NR2B-containing NMDA receptors, resulting in increased receptor internalization, and leading to a reduction in synaptic current (Chen et al., 2007; Li et al., 2009). Another result of increased GSK-3 activity is the phosphorylation of β-catenin and its subsequent degradation, leading to inhibition of GluN2B gene transcription (Li et al., 2009). Reports indicate that increased GSK-3 activity in might predispose synapses to LTD, suppress long-term potentiation (LTP), and/or shift the threshold of LTP induction through alteration of AMPA and/or NMDA receptor trafficking (Peineau et al., 2007, 2008; Bradley et al., 2012). Interestingly, it has been proposed that inositol depletion by lithium may disrupt LTP through its effects at IMPAs (Berridge et al., 1989), a hypothesis supported by evidence showing a lithium-induced reduction of cellular levels of IP3 (Berridge et al., 1989; Sarkar et al., 2005), a process critical to cytosolic calcium influx and LTP. In addition, inactivation of NMDA receptors of the dorsal hippocampus were proposed to be involved in lithium-induced spatial learning impairment (Parsaei et al., 2016). On the other hand, COX inhibitors have been demonstrated to have positive effects on HIP and cortex-related cognitive functioning in mice (Syed et al., 2015), but inhibit spatial memory retention in rats (Sharifzadeh et al., 2005) suggesting that the interplay between these different lithium targets are complex and make the resulting behavioral outcomes difficult to predict.

One notable similarity between SB 216763 and lithium was that administration of either drug increased HIP and PL theta oscillations, although only SB 216763 increased HIP–PL theta coherence. Theta rhythms are critically associated with movement (Vanderwolf, 1969), are present during behaviors associated with salient stimuli (Tesche and Karhu, 1999), and have been linked to behaviors related to decision-making processes, such as spatial memory in rodents (Jones and Wilson, 2005) or working memory in humans (Kahana et al., 1999; Tesche and Karhu, 1999; Raghavachari et al., 2001). Both repeated SB 216763 or lithium induced a 10 Hz HIP theta spike, although an overall increase in theta power was evident only following SB 216763 administration. The theta rhythm in HIP specifically is known to play a pivotal role in learning and memory function and to impact directly on performance in specific cognitive tasks (see reviews, Harris and Gordon, 2015; Korotkova et al., 2017). For example, disruption of the HIP theta rhythm in rats suppressed initial learning in the water maze (McNaughton et al., 2006), and reduced both working and reference memory performance in a continuous conditional discrimination task (Givens and Olton, 1994). Repeated SB 216763 and lithium administration were also shown to result in increased theta oscillatory activity in PL. Although less is known regarding the role of PFC theta in learning and memory, in human patients mid-frontal cortical theta activity was reported to serve as temporal context for the coordination of networks during learning, specifically within the medial PFC (Mas-Herrero and Marco-Pallares, 2016), and was also suggested to be integral in the regulation of ‘cognitive control’ (Cavanagh and Frank, 2014). SB 216763 increased HIP-PL theta coherence, although a similar change was not observed with lithium treatment. Studies have demonstrated links between HIP–PFC theta coupling with performance in a variety of spatial memory tasks (Jones and Wilson, 2005; Benchenane et al., 2010; Hyman et al., 2010).

A note on methodology: a caveat of the present study was that recordings were taken following water maze training with the rats anesthetized at the time of recording. The study was designed in this manner for two reasons. Firstly, the numerous conflicting reports in evaluating the impact of GSK-3 inhibition on learning and memory in healthy animals indicated we utilize a test in which the animals would be very motivated to complete and at the same time minimize variables that would contribute to alterations in neuronal oscillations, such as food restriction. In line with this, anesthetizing the animals would minimize contributions of neuronal oscillatory changes resulting from stress, fatigue, and consequently movement and alertness, and thus the focus would be predominantly on the exploration of direct drug effects. Clearly further characterization of GSK-3 effects on oscillations and their correlation to behavioral output would require recordings to occur in awake freely behaving animals. In addition, it is important to keep in mind that normalization of the spectra conserves the total power across the entire spectrum. Thus, what is represented are not changes in absolute values, but rather changes in the shape of the curve following drug treatment. Yet, despite these limitations, these findings lay the groundwork for a potential new avenue of research evaluating in depth the relationship between GSK-3 activity and neuronal oscillations in specific regions of the brain and the importance of this relationship in central nervous system disorders.

## Conclusion

The present findings point to a previously unknown role for GSK-3 in the regulation of neuronal HIP and PL theta oscillations in brain that could provide a lead into what could happen if GSK-3 activity became abnormally increased in a pathophysiologic state. Although conflicting reports of the therapeutic effects of lithium on cognition currently raise caution as to the therapeutic benefit of this drug in cognitive dysfunction disorders, furthering our understanding of the exact mechanisms that underlie the disparate effects of lithium on learning and memory would clearly be beneficial given its widespread use in the treatment of neuropsychiatric disorders. This idea is strengthened by the present findings which showed that direct inhibition of GSK-3 improved spatial memory and enhanced theta oscillations in both HIP and PL, two regions integral to cognitive function.

## Author Contributions

MP assisted with the surgeries, designed and performed the experiments, analyzed the data, prepared the figures and wrote the manuscript. TN and TF performed the surgeries and the experiments. SG contributed to the writing of the manuscript.

## Conflict of Interest Statement

The authors declare that the research was conducted in the absence of any commercial or financial relationships that could be construed as a potential conflict of interest.
