# Supplementary material for: Disparate Effects of Lithium and a GSK-3 Inhibitor on Neuronal Oscillatory Activity in Prefrontal Cortex and Hippocampus
Source: Front Aging Neurosci. 2018 Jan 12;9:434. doi: 10.3389/fnagi.2017.00434 (PMC5770585; doi:10.3389/fnagi.2017.00434)
Supplement: Supplementary file 1 [file Image_1.PDF]

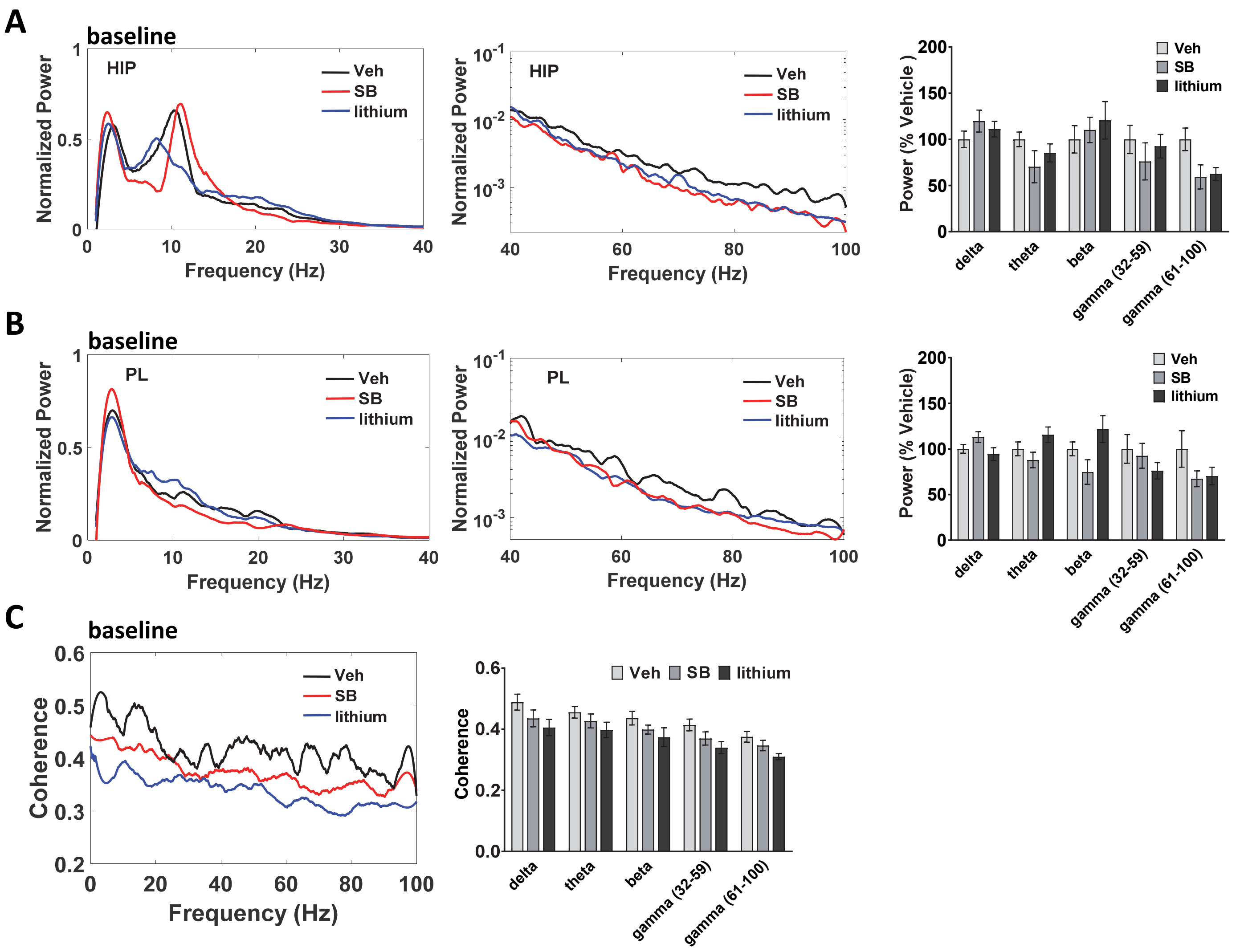

Fig S1. No difference between treatment groups as baseline. A, B) No significant group differences in spectral power were evident in HIP or PL at baseline. **C**) No significant group differences in HIP-PL coherence at baseline. N=8 rats/group with 1-2 recordings/region/rat. Curves represent group means following acute (red) and repeated (blue) drug administration. Bars represent means ± sem.
